# Supplementary material for: Momentum space toroidal moment in a photonic metamaterial
Source: Nat Commun. 2021 Mar 19;12:1784. doi: 10.1038/s41467-021-22063-w (PMC7979886; doi:10.1038/s41467-021-22063-w)
Supplement: Supplementary file 1 — Supplementary Information [file 41467_2021_22063_MOESM1_ESM.pdf]

Supplementary Information: Momentum Space Toroidal Moment in a Photonic Metamaterial

Biao Yang, Yangang Bi, Rui-Xing Zhang, Ruo-Yang Zhang, Oubo You, Zhihong Zhu, Jing Feng, Hongbo Sun, C. T. Chan, Chao-Xing Liu, Shuang Zhang

Supplementary Note 1 - Hybrid Wannier center and Berry curvature calculation

Closed Wilson loops are used to calculate both Wannier center and Berry curvature. Two bands (the 1st and 2nd bands) are considered as they are degenerate on the Brillouin boundary due to nonsymmorphic symmetries (two glide mirror symmetries in the $x-y$ plane). Supplementary Figure 1a shows the hybrid Wannier center calculation via $e^{i\Phi}=\det[W(L)]$ with Wilson operator $W(L)$ defined as,

$$W^{ij}(L)=\left\langle u_{k_0}^{i}\middle|\prod_{k_{\bar{\alpha}}\in L}\left(\sum_m |u_{k_\alpha}^{m}\rangle\langle u_{k_\alpha}^{m}|\right)\middle|u_{k_0}^{j}\right\rangle \quad (1)$$

where $i/j/m$ indicates the band index and $k_{\bar{\alpha}}$ is the point along the loop L with the bar over α indicating path ordering. We introduce the periodic gauge as shown in Supplementary Figure 1a to enable a closed loop for each (k_x, k_y) along k_z.

Supplementary Note 2 - Data post-processing

Usually, the Fourier transformation is carried as,

$$\mathcal{F}\left[A(f,r)\mathrm{e}^{i\phi(f,r)}\right] \quad (2)$$

where f and r indicate frequency and real position, respectively.

Supplementary Figures 4a and b show the corresponding results. As the field amplitude (Supplementary Figures 4a and b) is determined by transmission, loss and scattering, we also use the phase information independently to do the Fourier transformation as,

$$\mathcal{F}\left[1\mathrm{e}^{i\phi(f,r)}\right] \quad (3)$$

The trimmed results are shown in Supplementary Figures 4c and d (the same to Figs. 3f and i in the main text), where the interface states are much cleaner and sharper. We can understand the difference from Supplementary Figures 4e and f, where the amplitude decays away from the source position, and thus significantly broadening the interface states.

Supplementary Note 3 - Surface states with PEC/PMC boundary conditions

We carry out a surface state simulation for the nodal line configuration as shown in Supplementary Figure 8. There is no surface state for both PEC (perfect electric conductor) and PMC (perfect magnetic conductor) boundary conditions (The two boundary conditions are very common in photonics and are analogous to hard boundaries in condensed matters). Due to the finite size effect of the supercell, there is a small gap as illustrated by solid lines in Supplementary Figure 8a. The dashed lines indicate nodal line calculated with a single unit cell with imposing periodic boundary conditions along all x, y and z directions.

We perform a similar simulation as shown in Supplementary Figure 9 for the momentum-space toroidal moment (MTM). We find when setting the front and back surfaces to be PEC and PMC boundary conditions, respectively, the front and back surfaces support helical surface states. Here the PEC/PMC boundary condition serving as mirror ($M = -1/+1$) provides the other half Chern number[1]. This finding shows the vast difference between the system with MTM and that with nodal line.

Supplementary Note 4 - Effective media parameters

The effective media can be well described by the following constitutive matrices as ($\mathbf{D} = E\mathbf{E} + i\Gamma\mathbf{H}$ and $\mathbf{B} = M\mathbf{H} - \mathrm{i}\Gamma^{\mathrm{T}}\mathbf{E}$),

$$E = \begin{pmatrix} \epsilon & 0 & 0 \\ 0 & \epsilon & 0 \\ 0 & 0 & 1 \end{pmatrix}, M = \begin{pmatrix} \mu & 0 & 0 \\ 0 & \mu & 0 \\ 0 & 0 & 1 \end{pmatrix}, \Gamma = \begin{pmatrix} 0 & \gamma & 0 \\ -\gamma & 0 & 0 \\ 0 & 0 & 0 \end{pmatrix} \tag{4}$$

with,

$$\epsilon = 1 + \frac{l^2}{L(\omega_0^2 - \omega^2)}, \mu = 1 + \frac{A^2\omega^2}{L(\omega_0^2 - \omega^2)}, \gamma = \frac{Al\omega}{L(\omega_0^2 - \omega^2)} \tag{5}$$

where l, A , L, ω_0 are effective length, area, inductance and resonance frequency, respectively. In order to introduce non-local effect, here we set,

$$l = 1 - \frac{a(k_x^2 + k_y^2)}{b + c(k_x^2 + k_y^2)} \tag{6}$$

In all of calculations, we set $a = 1, b = 10, c = 1, L = 1, \omega_0 = 2, A = 1/20$ for MTM and $A = 0$ for nodal line, respectively.

An intuitive understanding relating the phenomena (shown in Fig. 5 of main text) to the Berry curvature distribution can be made as following. For the brevity of analytical results, here we focus on one valley with Hamiltonian as $H_v = k_z\sigma_x + \gamma\sigma_y + \delta k_x\sigma_z$, where we set $k_y = 0$ to be the reflection plane and $z = 0$ ($z < 0$ is occupied by the valley material) as the reflection interface. When the incident energy (frequency) $E = 0$, no mode can propagate in the valley material and thus one sees total reflection. The corresponding eigenstates carry all of the topological information, with $|u\rangle = \left(i\frac{\gamma + \sqrt{\delta k_x^2 + \gamma^2}}{\delta k_x}, 1 \right)$, where $k_z = -i\sqrt{\delta k_x^2 + \gamma^2}$. We assume $\gamma > 0 (\gamma < 0)$ corresponds to the front/back surface. Considering the limit $\lim_{\delta k_x \to \pm\infty} |u\rangle = (\pm i, 1)$, which is independent of the value of γ, one can construct a closed loop consisting of the following two paths: 1, on the front surface δk_x runs from $-\infty$ to ∞; 2, on the back surface, δk_x runs from $+\infty$ to $-\infty$. Along this close loop, the eigenstate $|u\rangle$ travels on the Poincaré sphere and constructs a closed loop as well, which subtends a solid angle of $\text{sgn}(\gamma)\pi$. It clearly implies the tight relation to valley Chern number-$\text{sgn}(\gamma)$ or quantized Berry

curvature with the integral flux being $\mathrm{sgn}(\gamma)\pi$. Finally, the reflection phases with the same incident wave are uniquely determined by the eigenstates $|u\rangle$ via boundary conditions. In the metamaterials, the front surface having $|u\rangle = (1,0)$ at $\delta k_x = 0$ behaves like PEC (in-plane electric field is 0), thus one expects the π phase jumping. While the back surface ($|u\rangle = (0,1)$ at $\delta k_x = 0$) being similar to PMC (out-plane electric field is 0) shows just a slight reflection phase change. Following the closed loop defined above, the total phase increases $2\pi - \Delta + \Delta = 2\pi$.

Reference:

1 Yao, W., Yang, S. A. & Niu, Q. Edge States in Graphene: From Gapped Flat-Band to Gapless Chiral Modes. Physical Review Letters 102, 096801, doi:10.1103/PhysRevLett.102.096801 (2009).

Figures:

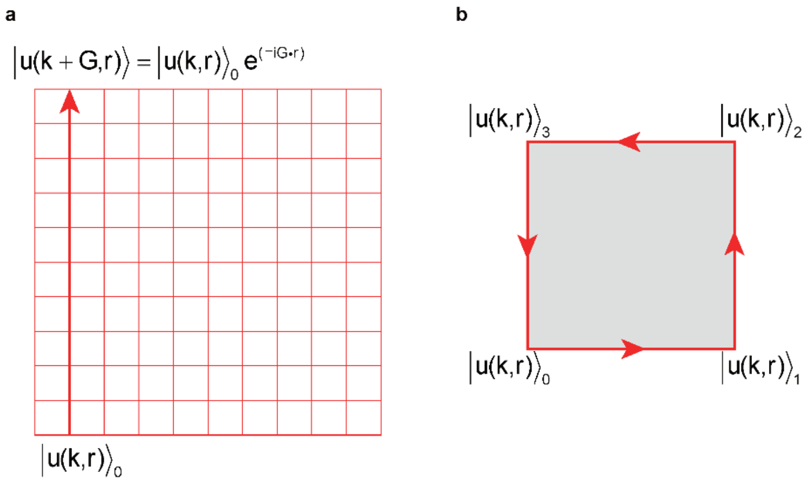

Supplementary Figure 1| Wannier center and Berry curvature calculation based on Wilson loops. a, Wilson loop along the red arrow is calculated, where the periodic gauge is considered. We used 90 points to cover the path ΓXMΓ. Along $k_z \in [-180, 180]$, 31 points are calculated. **b,** Berry curvature calculated based on the small closed Wilson loops. We used 35 and 37 cells along $k_x \in [5, 175]$ and $k_z \in [-180, 180]$, respectively. In both cases, the two bands (below gap) are considered.

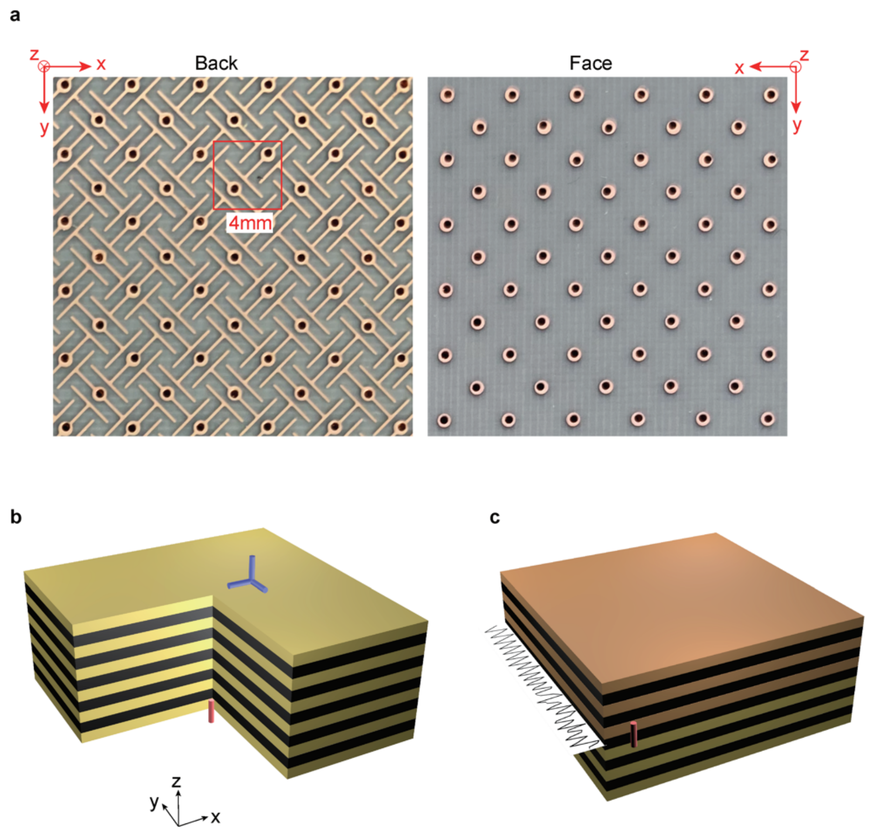

Supplementary Figure 2| Experimental setup for mapping bulk and Back-Back/Face-Face interface states. a, Sample fabricated by printed circuit board (PCB) in a commercial company. Face and back sides are defined. **b,** Setup for probing bulk states. The source antenna (red) is located beneath the center of the bottom layer. The component of probing electric field can be controlled by adjusting the orientation of the probing antenna (purple). We used 20-unit cells when measuring bulk states. In order to show the source antenna, one quarter sample is cut. **c,** Interface state mapping with line-scanning the interface constructed by 10 vs 10-unit cells. Source antenna is indicated in red.

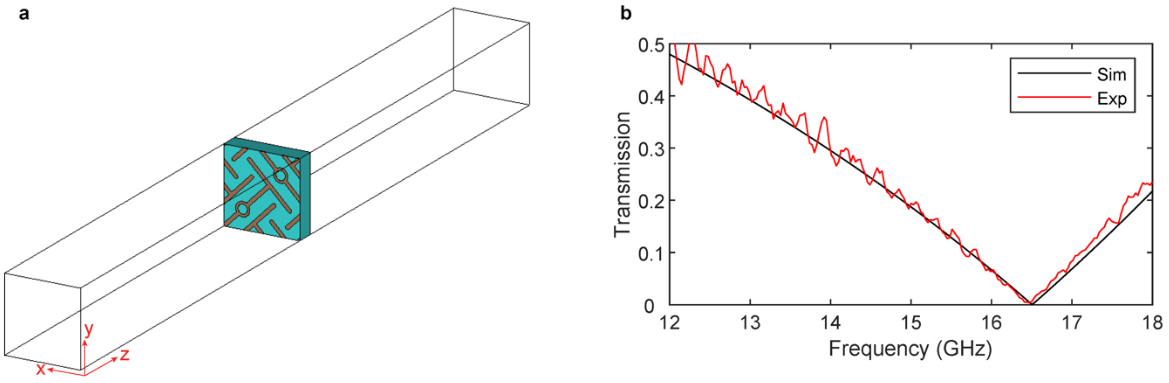

Supplementary Figure 3| Transmission spectrum for one single layer. a, Simulation configuration in CST microwave studio. The single sample layer is surrounded by air with dielectric constant of 1. The permittivity of hosting material is set to be 1.8 aiming to fit the transmission resonance dip as shown in the panel (b). **b,** Experimentally measured and theoretically simulated transmission resonance dips ($|\frac{E_{TM}^{out}}{E_{TM}^{in}}|$). The experimental result is normalized with a reference setup without sample.

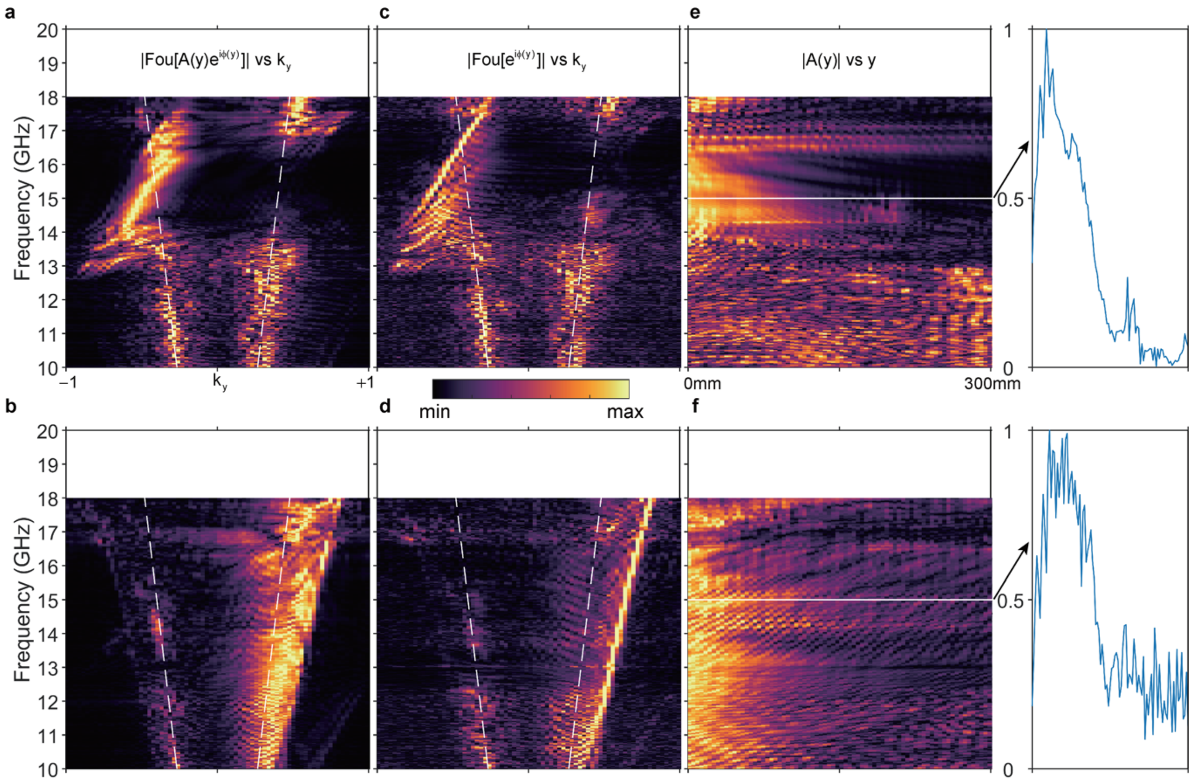

Supplementary Figure 4| Back-Back/Face-Face interface states under different post data-processing. a/b, Fourier transformation considers both amplitude and phase for the Back-Back/Face-Face interface state. **c/d,** Fourier transformation considers only phase for the Back-Back/Face-Face interface state. **e/f,** Amplitude decaying along y direction for the Back-Back/Face-Face interface state. The insets show the amplitude decaying at 15 GHz.

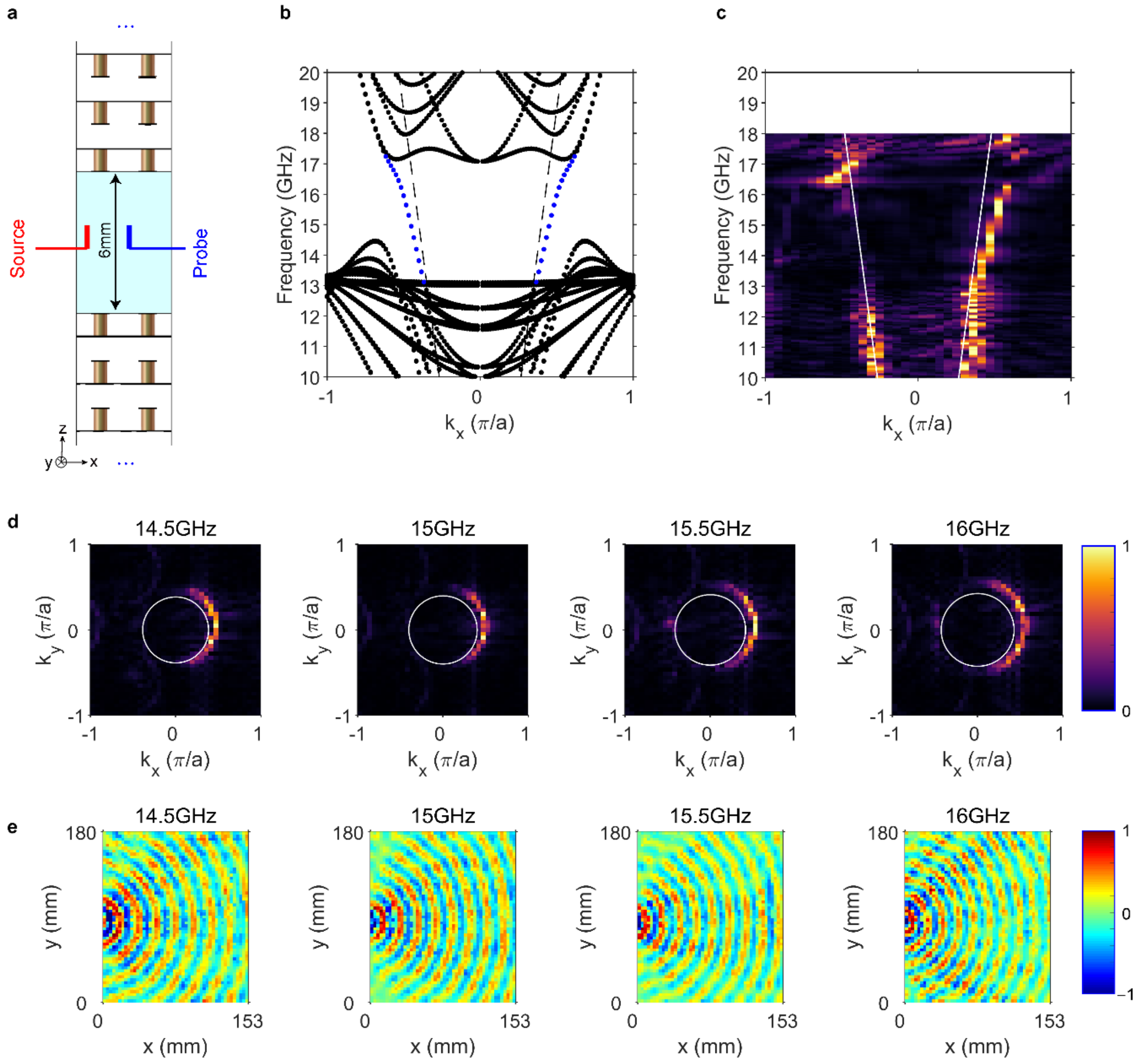

Supplementary Figure 5| Face-Face air-gap interface state scanning. a, Configuration for both simulation and experiment scanning. An air-gap with thickness of 6 mm is built to raster-scan the Face-Face interface states. Source and probe antennas are schematically illustrated. **b/c,** Numerically simulated/experimentally probed interface states along k_x . **d/e,** Momentum/real space interface states at different frequencies.

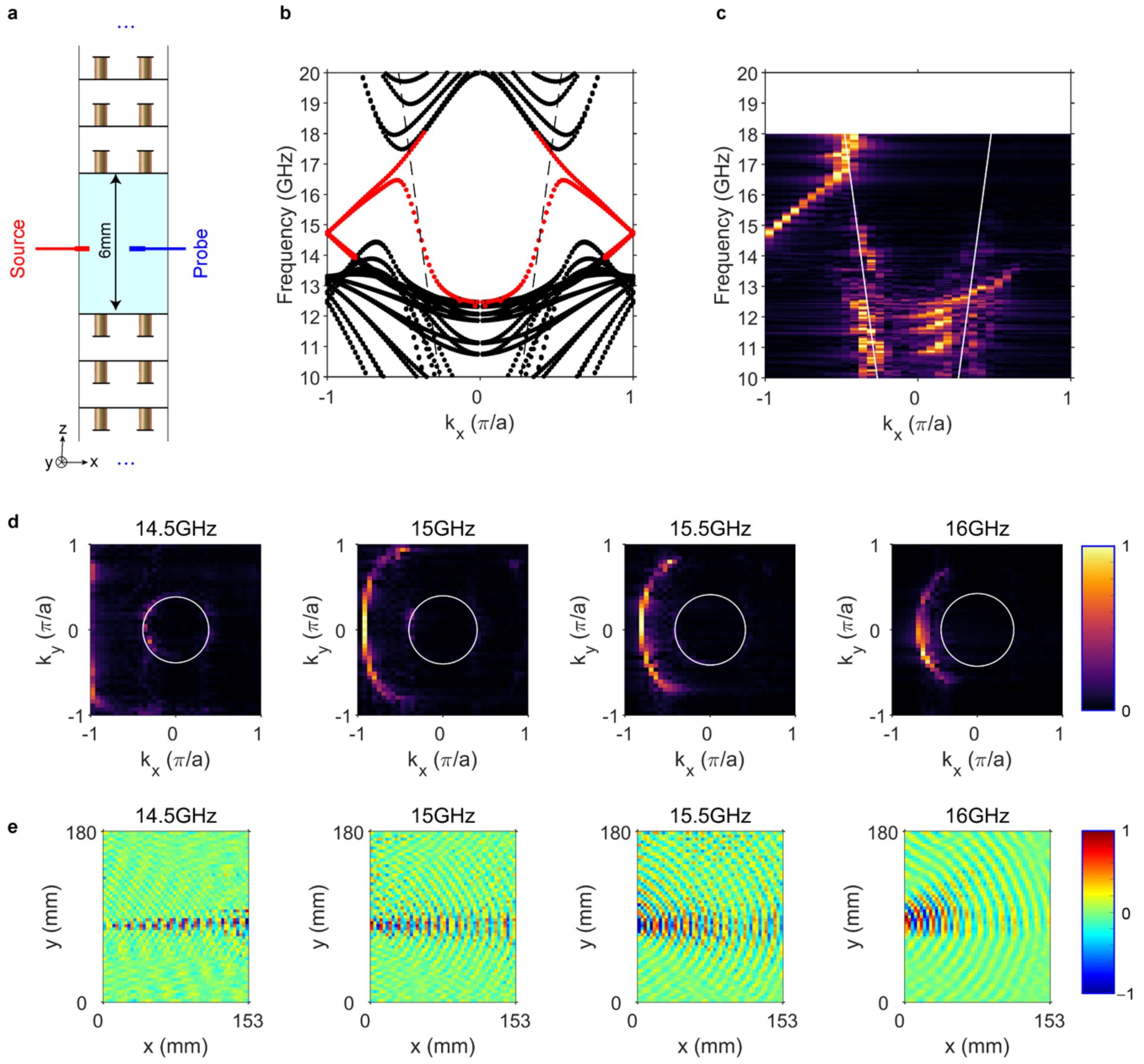

Supplementary Figure 6| Back-Back air-gap interface state scanning. a, Configuration for both simulation and experiment scanning. An air-gap with thickness of 6 mm is built to raster-scan the Back-Back interface states. Source and probe antennas are schematically illustrated. **b/c,** Numerically simulated/experimentally probed interface state along k_x . **d/e,** Momentum/real space interface states at different frequencies.

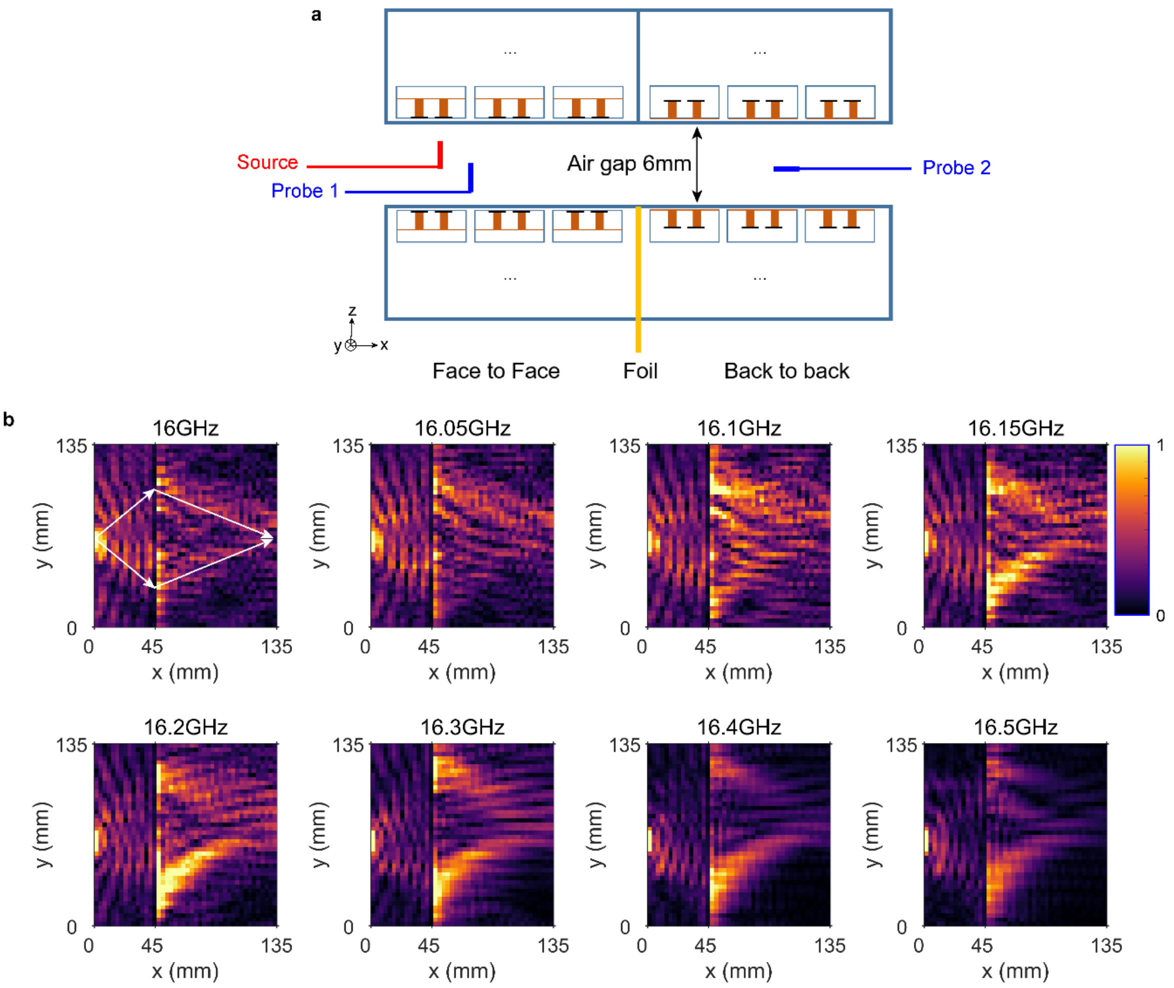

Supplementary Figure 7| Negative refraction of two-dimensional interface states. a, Configuration used to characterize the negative refraction phenomena. The Face-Face/Back-Back configuration supporting positive/negative refraction index is located on left/right-hand side. The source antenna is located on the leftmost, two different probe antennas are used to scan the interface (or guiding) waves. The copper foil is used to break mirror symmetry on the interface and thus Face-Face interface states can excite Back-Back interface states across the sharp domain-wall ($x = 45$ mm in (b)). **b,** Negative refraction phenomena at different frequencies. Here the negative refraction effect enables perfect lens, i.e., bringing the light from a point source (on the left-hand side) to a focal point on the right-hand side.

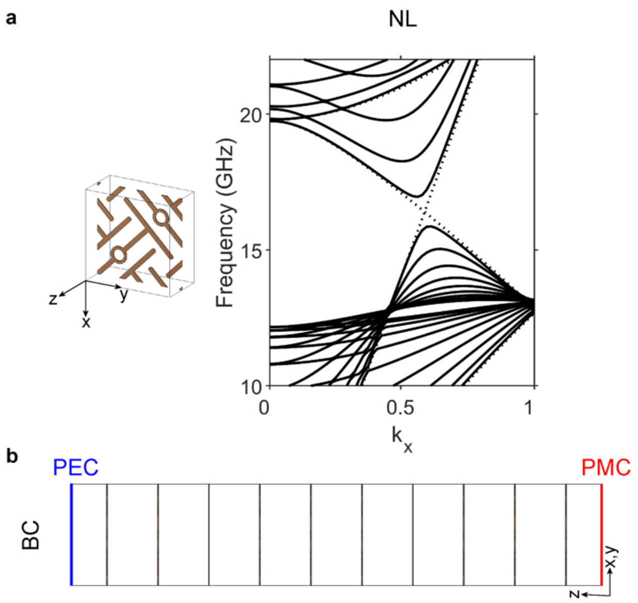

Supplementary Figure 8| No surface states for the nodal line (NL) configuration with both PEC (perfect electric conductor) and PMC (perfect magnetic conductor) boundary conditions (BC). The solid lines indicate the bands calculated with the supercell shown in (b). There is a small gap in the middle due to the finite size effect of the supercell. Dashed lines are the bands of nodal line calculated by imposing periodic boundary conditions along all x, y and z directions on a single unit cell (inset in (a)).

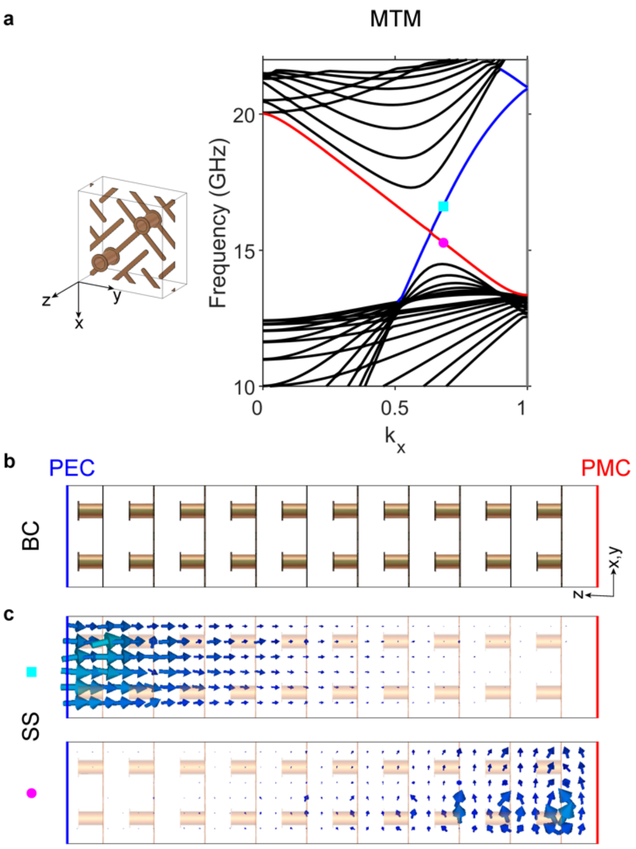

Supplementary Figure 9| Surface states (blue and red) supported by momentum-space toroidal moment (MTM) with PEC (perfect electric conductor) and PMC (preface magnetic conductor) boundary conditions on the front and back surfaces, respectively. a, Calculated surface states. **b,** Supercell with PEC/PMC boundary condition (BC). **c,** The corresponding surface states (SS) as marked in (a).

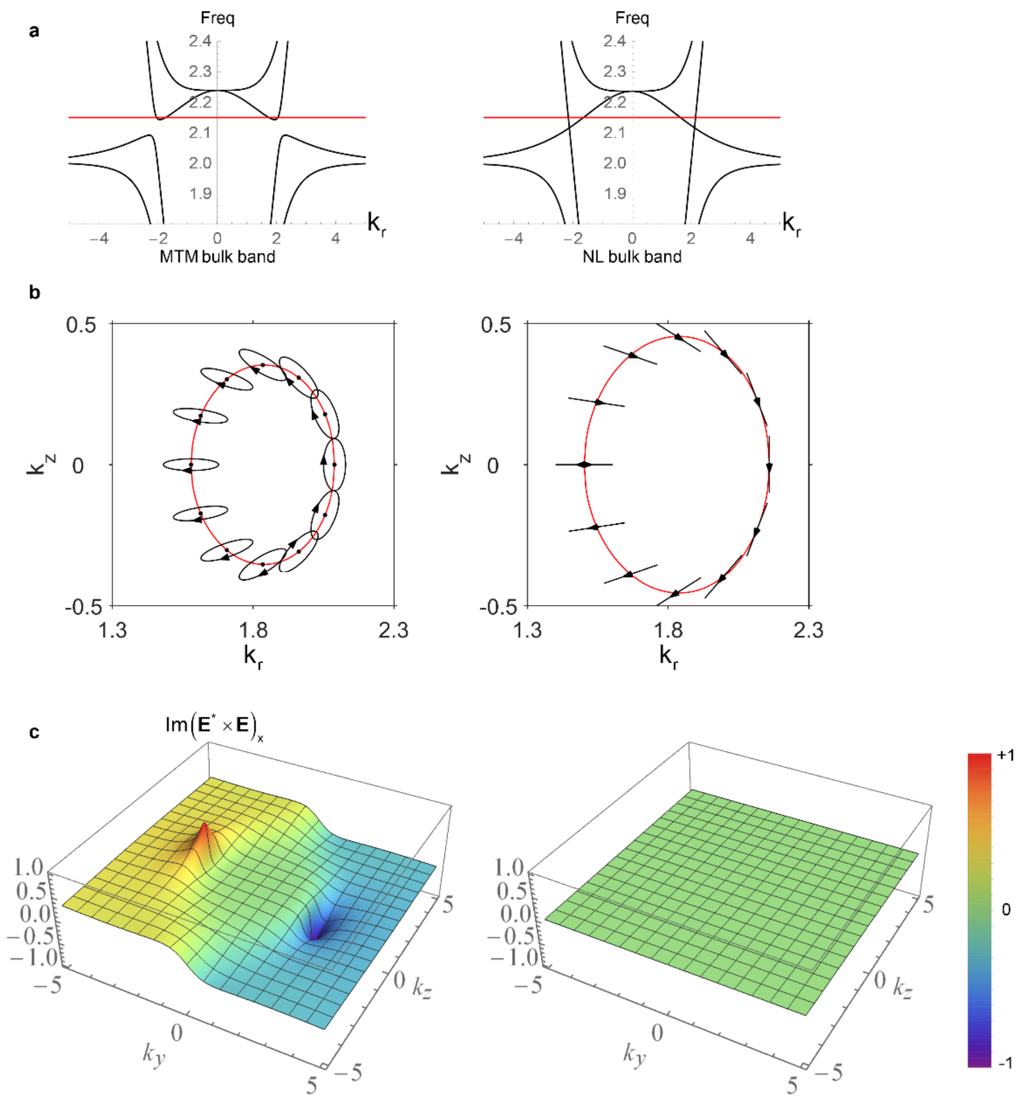

Supplementary Figure 10| Transverse spin in the momentum-space toroidal moment (MTM). a, The bulk band structure of the MTM and nodal line (NL). The red lines indicate the frequency used to plot the eigen electric fields. **b,** Elliptically and linearly polarized electric fields in MTM and NL correspondingly. In the MTM the elliptical electric field orbits induce transverse spins pointing in the plane. **c,** Transverse spin defined as $\mathrm{Im}(\mathbf{E}^* \times \mathbf{E})_x$, calculated with the 2nd band for both MTM and NL.
